# Localized Wilms’ tumor in low-middle-income countries (LMIC): how can we get better?

**DOI:** 10.1186/s43046-020-00043-3

**Published:** 2020-08-14

**Authors:** Hosam Y. Asfour, Sahar A. Khalil, Al-Shimaa Zakaria, El-Sayed Ashraf, Wael Zekri

**Affiliations:** 1 Pediatric oncology department, National Cancer Institute, Cairo University, and 57357 CCHE Hospital, Fom El-khalig Square, Kasr El-Aini St, Cairo, 11796 Egypt; 2 Pathology Department, National Cancer Institute, Cairo University and 57357 CCHE Hospital, Cairo, Egypt; 3 Surgical Oncology Department, National Cancer Institute, Cairo University, Cairo, Egypt

**Keywords:** Wilms’ tumor, Loco-regional, Egypt, Pediatric, Outcome

## Abstract

**Background:**

Wilms’ tumor (WT) represents about 6% of all childhood cancers. The overall survival markedly improved to exceed 90% in developed countries, yet some studies from developing counties still have poorer outcomes. The aim of this study is to assess the clinical outcome and the different prognostic factors that influence the outcome of pediatric loco-regional WT cases treated at National Cancer Institute (NCI), Cairo University, Egypt. This is a retrospective study which included pediatric loco-regional WT patients presented between January 2008 and December 2017. Patients were followed up till June 2019.

**Results:**

Ninety-two eligible patients were included. Median age was 3 years (range 1 month–9 years). Abdominal mass was the commonest presentation (72.8%). The 5-year EFS and OS of the whole group was 83.7% and 94.6% retrospectively. Despite having a similar EFS (84.8 vs. 82.6%), stage III patients had a significantly lower OS than those in stages I and II (89.1% vs. 100%, *p* value 0.024). Twelve patients had unfavorable histology and had a significantly lower EFS and OS than the patients with favorable histology (50 and 83.3% vs. 88.8 and 96.3%, *p* value < 0.001 and 0.043, respectively).

**Conclusion:**

Loco-regional Wilms’ tumor cases treated in Egypt had OS nearly the same as in developed countries, but had a lower EFS than expected mainly stages I and II. The stage and histological type are the main factors influencing the survival, and further studies are needed to investigate nuclear unrest grades and proper management of such cases.

## Background

Wilms’ tumor WT represents about 6% of all childhood cancers. Children with Wilms’ tumor are mostly healthy children with asymptomatic abdominal mass, discovered accidentally. Hematuria, abdominal pain, or malaise is found in 20 to 30% of cases. About 25% of cases could have hypertension [1]. Wilms’ tumor is associated with several genetic syndromes that can affect the clinical presentation. Approximately 5% of children who have Wilms’ tumor present with bilateral disease [2].

The two approaches that involve the diagnosis and treatment of Wilms’ tumor are the Children’s Oncology Group (COG) in North America and International Society of Paediatric Oncology (SIOP) in Europe. The COG approach favors upfront nephrectomy for precise staging and histological assessment [3], while the SIOP approach recommends a preoperative chemotherapy that will allow reducing tumor size and risk of intraoperative tumor spillage [4]. Radical nephrectomy with lymph node sampling is the procedure of choice [5]. Local staging of Wilms’ tumor is defined by the results of imaging studies, surgical details, and pathological findings.

The usual pathology of WT consists of variable proportions of 3 cellular components, namely, blastemal, epithelial, and stromal. Blastema represents the least differentiated and presumed most malignant component [6]. The tumors resected following chemotherapy with significant amounts of persisting blastema require more aggressive therapy [7].

Anaplastic WT accounts for 5–8% of all WT cases [6]. The anaplastic tumors are associated with a poor prognosis, especially at the higher stage [7]. Some Wilms’ tumors with favorable histology show disturbing nuclear enlargement, cytologic atypia but without multipolar mitotic figures, known as nuclear unrest [8]. Nuclear unrest is categorized as grades I, II, and III. Grade I has minimal disorder with nuclear diameter approximating that of red blood corpuscle (RBC), while grade III has striking cytologic atypia without multipolar mitotic figures. Grade II is intermediate [9]. There appears a higher risk of recurrence with unrest, but the overall prognosis appears to be the same as for non-anaplastic tumors [7].

The overall survival markedly improved to exceed 90% in developed countries, yet some studies from developing counties still have poorer outcomes [10]. The aim of the study was to describe the frequency and epidemiology of loco-regional Wilms’ tumor cases treated in Egypt, to assess treatment outcome regarding overall survival (OS) and event-free survival (EFS), and to evaluate different prognostic factors in relation to outcome with discussion of some problems that might influence the diagnosis and staging.

## Methods

This is a retrospective study, including all pediatric patients with loco-regional unilateral Wilms’ tumor (WT) (with favorable and unfavorable histology) at National Cancer Institute (NCI), Cairo University, Egypt from the 1st of January 2008 till the end of December 2017. Patients were followed up till the end of June 2019.

Records of all patients were reviewed for demographic characteristics of the patients, investigations done (CTs, pathological reports), type of surgery, treatment received (radiotherapy, and chemotherapy), response to treatment (according to Response evaluation criteria in solid tumors (RECIST) criteria version 1.1 [11]), and treatment-related toxicities (according to Common Terminology Criteria for Adverse Events (CTCAE) version 4.0 [12]). Tumor staging and pathology were revised using COG clinic pathologic staging of Wilms’ tumor [1].

The initial diagnosis was done with CT abdomen to check the site and nature of the renal mass and for liver metastasis and chest CT to exclude lung metastasis. Surgical consultation regarding upfront nephrectomy was done for all patients. Upfront guided biopsy percutaneous using trucut needle 12 gauge was done in locally advanced patients who were refused to do upfront nephrectomy. No patients underwent open biopsy.

Wilms’ tumor patients were treated according to the COG strategy with some modifications. According to the COG approach, stage I and II favorable histology (FH) patients are treated by chemotherapy consisting of vincristine and dactinomycin without radiotherapy. Stage III patients have a postoperative residual tumor confined to the abdomen. This could be in the form of gross or microscopic residual, tumor spillage, peritoneal deposits, inferior vena cava (IVC) thrombus, lymph node (LN) involvement, or tumor biopsy. A combination of dactinomycin, vincristine, doxorubicin, and 10.8 Gy of radiation therapy to the flank is used to treat stage III FH patients, and whole abdominal irradiation is indicated for extensive intraperitoneal disease or widespread intraperitoneal tumor spill [1, 13].

The institutional modifications of COG protocol were as follows: no group is considered as a very low risk group and all patients received adjuvant chemotherapy, loss of heterozygosity (LOH) 1p and 16 q was not considered for risk stratification, patients that were considered irresectable upfront underwent guided biopsy (no patient had open biopsy, or exploration), and all unilateral patients who underwent delayed nephrectomy after receiving chemotherapy were checked for blastemal predominance to consider intensifying chemotherapy.

Blastemal predominance was diagnosed after giving neoadjuvant chemotherapy and defined as presence of more than one third of the tumor viable, and more than two third of this viable tumor is blastemal component [7]. The anaplasia was diagnosed by the presence of 3 criteria, atypical tri- and multipolar mitotic figures, marked nuclear enlargement, and hyperchromatism. The anaplasia was considered a diffuse anaplasia if it was multi-focal anaplasia, anaplasia in an extrarenal site, and in a random biopsy and had presence of focal anaplasia and marked nuclear unrest elsewhere in the tumor [7]. All pathological sections of nephrectomies with focal anaplasia were examined to search for other foci of anaplasia. Wilms’ tumors with nuclear unrest contain tumor cells with enlarged, hyperchromatic nuclei but do not have the enlarged, multipolar mitotic figures required to meet the criteria for anaplasia [9]. Nuclear unrest did not influence the choice of treatment protocol, but was assessed retrospectively for the outcome of disease.

Flank radiotherapy was indicated for all stage III patients and anaplastic stage I and II patients. The patients with diffuse tumor spillage needed whole abdominal irradiation. The dose of radiotherapy was 10.8 Gy except for stage III patients with diffuse anaplasia who needed to receive 19.8 Gy except if infant.

Event-free survival EFS is defined as the time from date of diagnosis to date of relapse, progression, death, or the last follow-up date. Overall survival (OS) is defined as the time from date of diagnosis to date of death or last follow-up. A *p* value < 0.05 was considered significant.

## Results

**Fig. 1:**
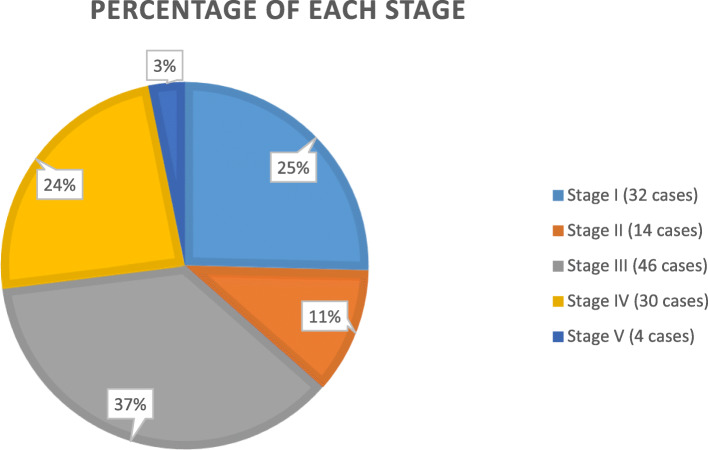
Percentage of each stage

This is a retrospective cohort study, conducted at the National Cancer Institute (NCI), Cairo University, Egypt, including all pediatric patients diagnosed as having loco-regional Wilms’ tumor (WT) (*n* = 92) from a total of 126 WT patients presented during the period from the 1st of January 2008 to the end of December 2017. Thirty patients have been excluded from the study being metastatic, and 4 patients had bilateral Wilms’ tumors (Fig. 1, Table 1).

**Table 1:** Demographic and clinical characteristics of studied patients

		Number	Percent
Gender	Male	62	67.3%
	Female	30	32.6%
Laterality	Right	46	50%
	Left	46	50%
Common disease presentations	Abdominal mass	67	72.8%
	Hematuria	13	14.1%
	Abdominal pain	12	13%

### Diagnosis

Our institutional policy regarding new patients with renal mass is to order abdominal CT to check the nature of the mass and its extensions and search for hepatic metastasis, and chest CT to check for lung metastasis. Surgical consultation regarding upfront nephrectomy was done for all patients.

**Fig. 2:**
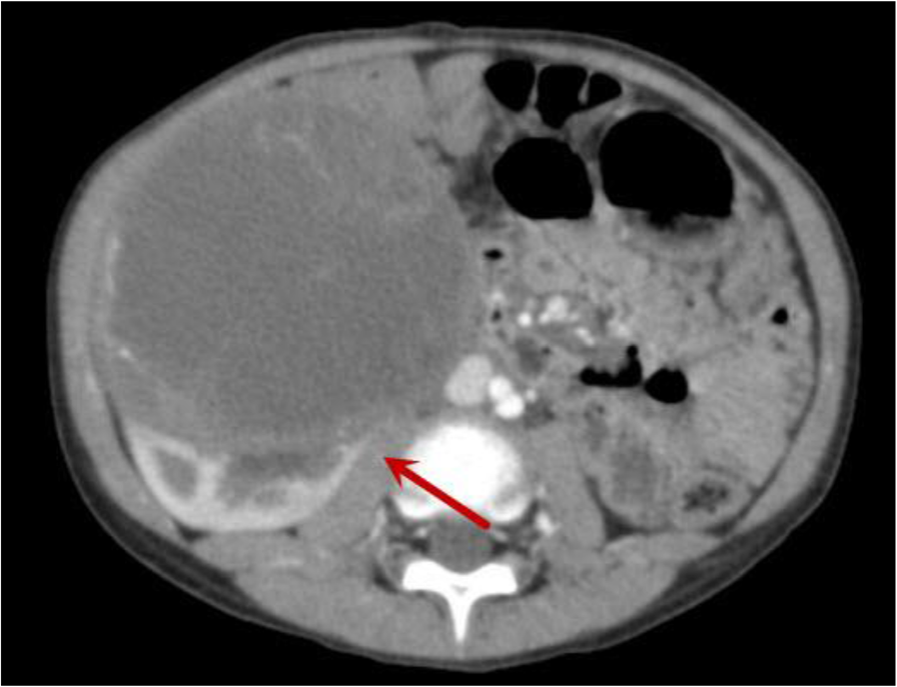
CT abdomen showing right Wilms’ tumor with claw sign (red arrow)

**Fig. 3:**
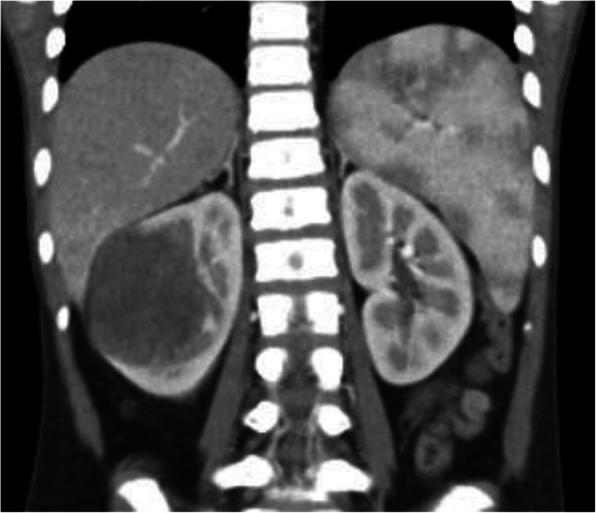
CT abdomen showing right small Wilms’ tumor. The case underwent upfront nephrectomy after surgical consultation

**Fig. 4:**
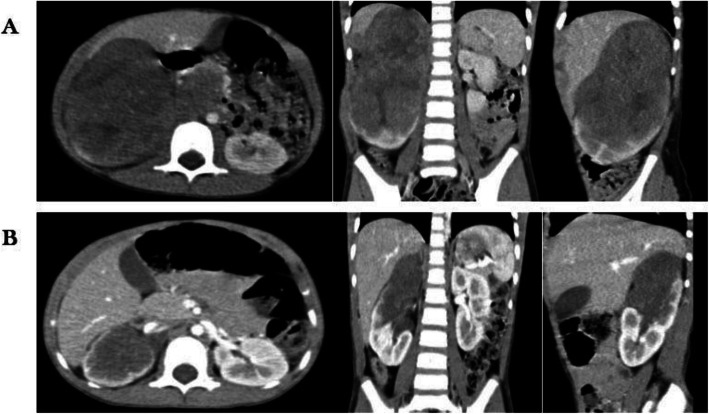
CT abdomen showing right Wilms’ tumor locally advanced. **a** Initial imaging at presentation. **b** Imaging post 6 weeks of chemotherapy showing regressive course

The average of the largest diameter of the renal masses in the initial CTs was 11.20 cm (range from 2.7 to 33 cm). The average of the largest diameter was higher in advanced stages. Stage I patients had an average largest diameter of 9.39 cm, stage II patients had an average largest diameter of 10.90 cm, and stage III patients had an average largest diameter of 12.43 cm (Figs. 2, 3 and 4).

### Nephrectomy and LN sampling

Sixty-three patients underwent upfront nephrectomy (68.4%), and 29 patients were unresectable upfront and needed preoperative chemotherapy (31.5%). Twenty-nine patients in our study had initial biopsy, all of them was ultrasound guided.

One patient (1%) had preoperative tumor rupture, and 8 patients (8.6%) had intraoperative spillage with capsular rupture. The spillage incidence was higher during upfront nephrectomy (6 out of 63 patients, 9.5%) than that during delayed nephrectomy (2 out of 29 patients, 6.8%), and 5 of such tumors were right-sided.

Two patients developed intestinal obstruction during follow-up years. One of them was after the 8 months follow-up and needed surgical exploration, and the other patient was after 22 months follow-up and resolved on conservative measures.

**Fig. 5:**
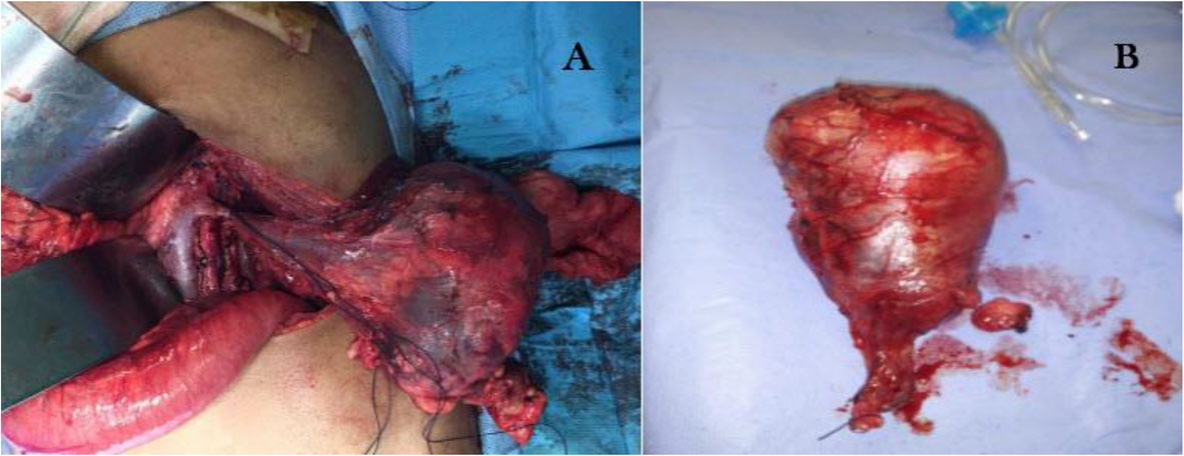
Left radical nephrectomy operation. **a** During the operation, **b** left kidney with LN sampling

Lymph node sampling was done in 49 patients (53.2%) (17 out of 46 patients with stages I and II (36.9%), 32 out of 46 patients with stage III (69.5%)). The median number of lymph nodes was 3 (ranging from 1 to 33 LNs). There were 43 patients who did not have LN sampling at the operation (Fig. 5).

### Histopathological examination

**Fig. 6:**
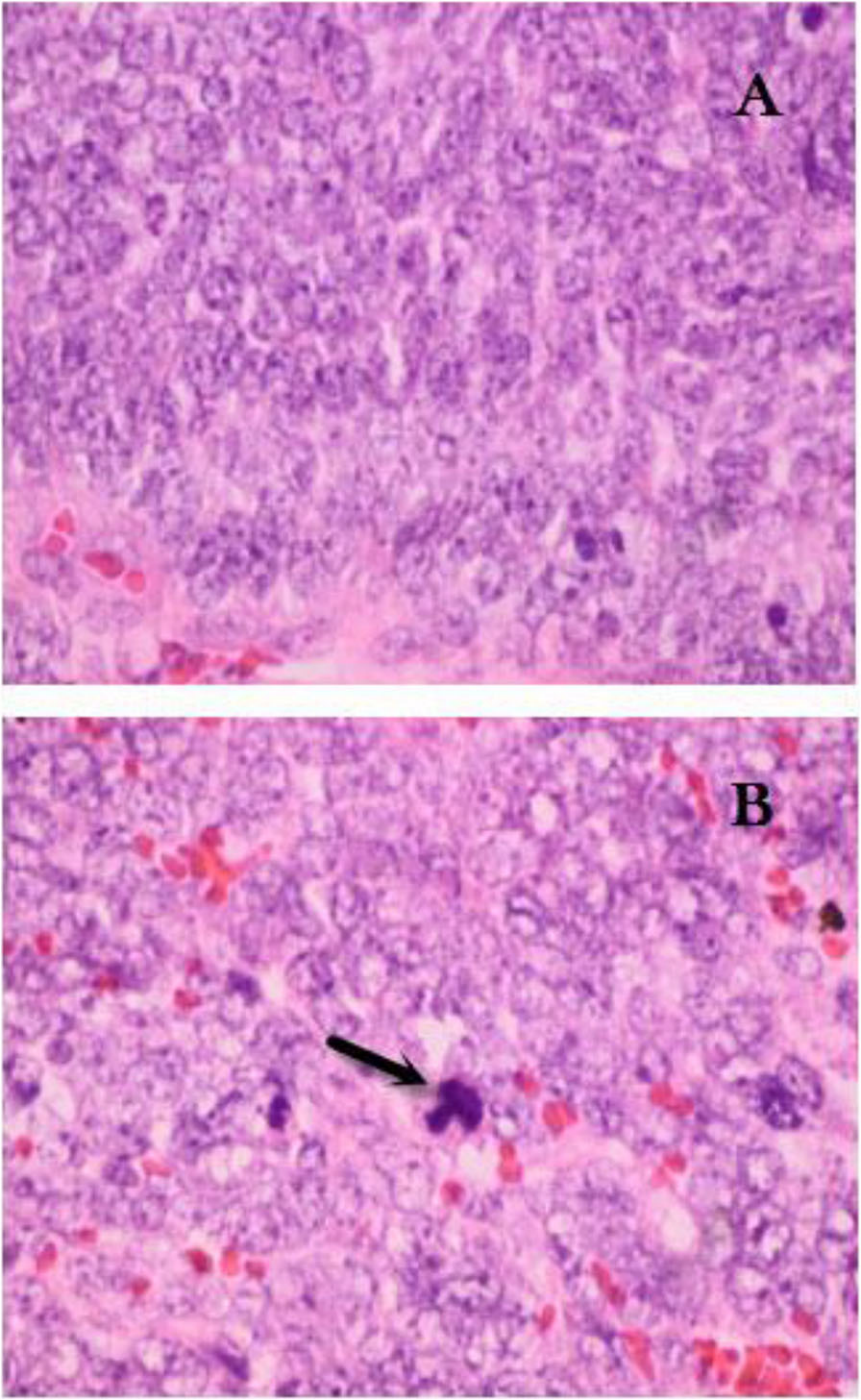
Two Wilms’ tumor cases. **a** Wilms’ tumor with nuclear unrest grade III showing enlarged, hyperchromatic nuclei but do not have the enlarged, multipolar mitotic figures. **b** Anaplastic Wilms’ tumor with atypical mitotic figures (black arrow), marked nuclear enlargement, and hyperchromatism

**Fig. 7:**
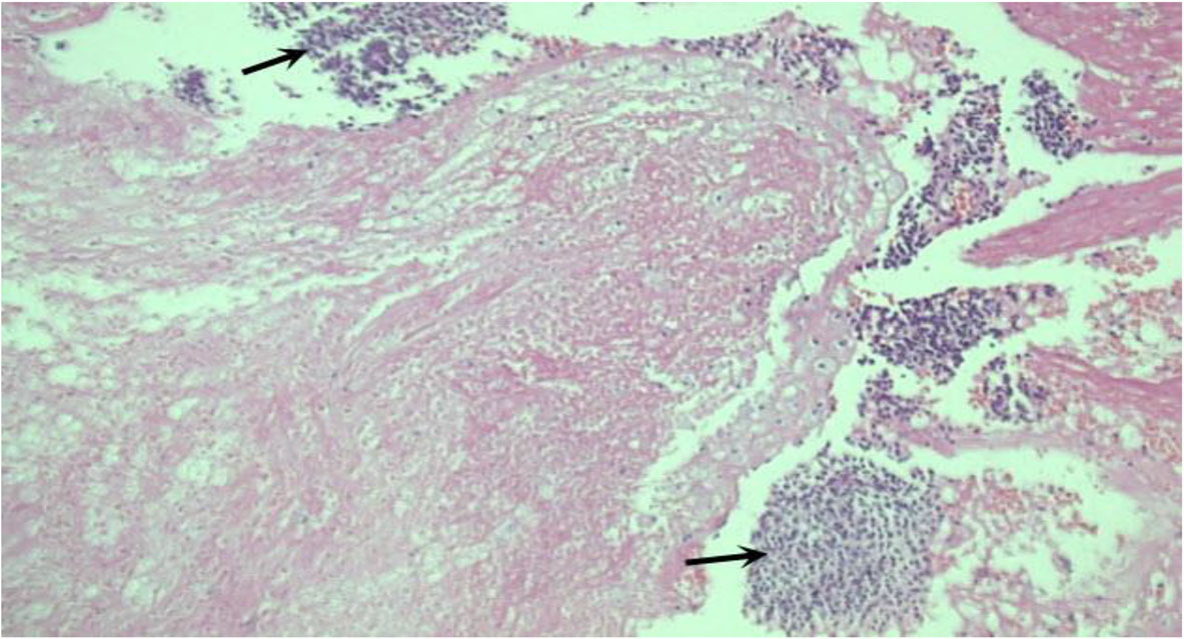
Showing marked therapy effect with necrosis and residual groups of blastemal components (arrow) (× 200)

During the study, the slides of the patients were revised to recheck the local stage and histopathological subtypes. Eighty patients (86.9%) had favorable histology, 4 patients had nuclear unrest (4.3%), 4 patients had blastemal predominance (4.3%), and 4 patients had anaplasia (3 was focal, and 1 case had diffuse anaplasia) (4.3%) (Table 2) (Figs. 6 and 7).

**Table 2:** Histopathological subtypes

Stage	Favorable	Nuclear unrest	Blastemal predominance	Anaplasia	Total
Stage I	30	1	0	1	32
Stage II	11	2	0	1	14
Stage III	39	1	4	2	46
Total	80	4 (4.3%)	4 (4.3%)	4 (4.3%)	92

**Fig. 8:**
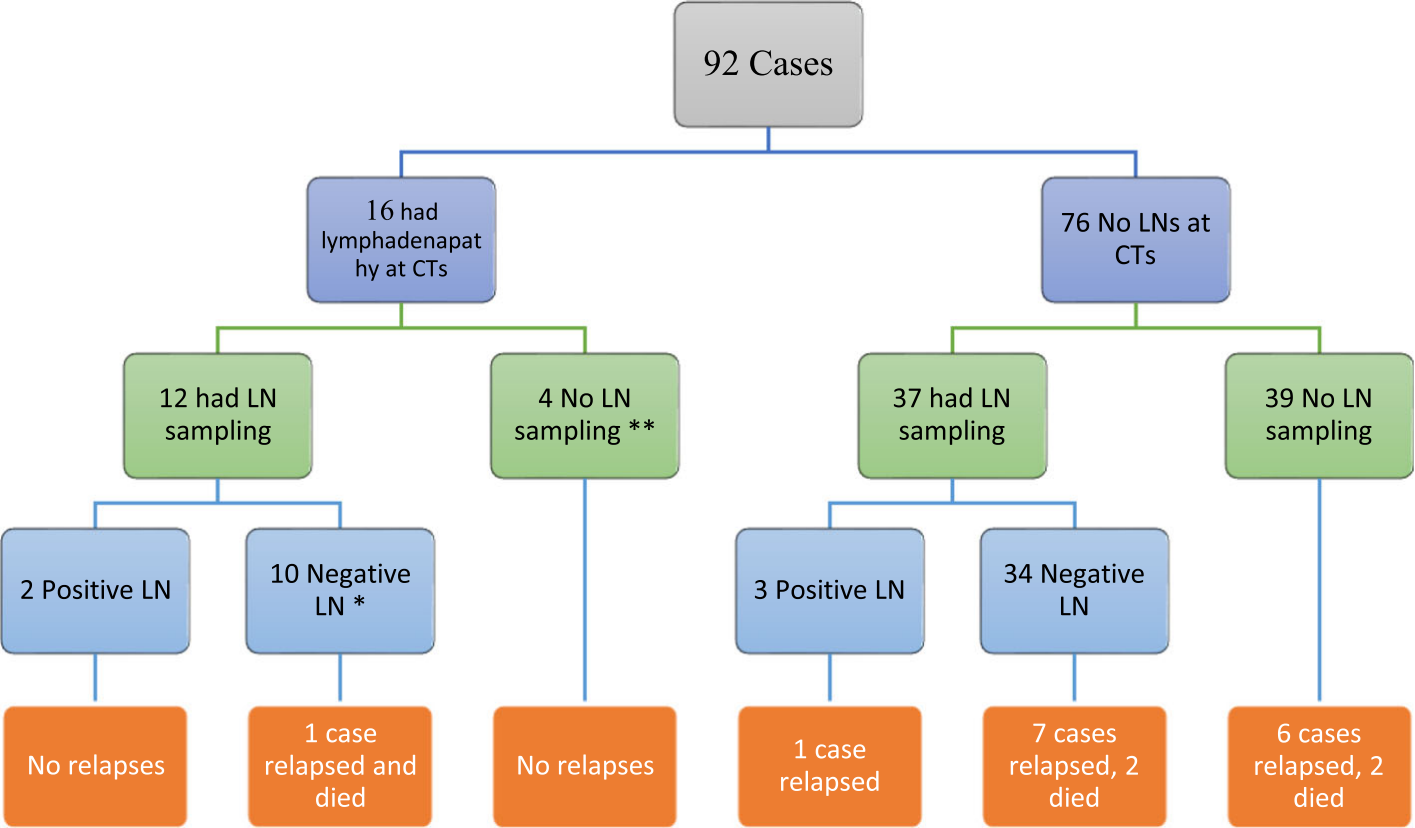
LN sampling and lymphadenopathy in initial CTs. *One case of them was considered LN positive as the nephrectomy was post-chemotherapy, **One case of them was considered LN positive being significant at CTs

After analysis of the 49 patients who had LN sampled during the operation and comparing the pathology results with the CT results, only 2 out of 5 patients with positive LN in pathology had lymphadenopathy in initial CT (sensitivity 40%) (Fig. 8 and Table 3).

**Table 3:** Gold standard test to assess CT ability to detect positive LN

			LN in pathology		Total
			Positive	Negative	
Lymphadenopathy in CT	Yes	Count	2	10	12
		% within CT	16.6%	83.3%	100.0%
		% in positive LN	40.0%	22.7%	17.8%
		% in total	4.4%	13.3%	17.8%
	No	Count	3	34	37
		% within CT	8.1%	91.9%	100.0%
		% in positive LN	60.0%	77.2%	82.2%
		% in total	6.7%	75.6%	82.2%
Total		Count	5	44	49
		% within CT	10.2%	89.8%	100.0%
		% in positive LN	100.0%	100.0%	100.0%
		% in total	10.2%	89.8%	100.0%
Sensitivity 40%		Specificity 77.2%		Positive predictive value 16.6%	
Negative predictive value 91.9%		False negative rate 60%		False positive rate 22.7%	

### Radiotherapy and chemotherapy

Stage I and II FH patients did not receive radiotherapy. Seven patients out of 46 stage III cases have not received radiotherapy. Of them, 4 patients were treated during 2008 when our oncology team treated stage III cases due to preoperative chemotherapy without radiotherapy, but they received DD4A regimen (three-drug regimen) used for stage III. One patient died out of sepsis post-chemotherapy before receiving radiotherapy. Two patients were delayed for radiotherapy till radiotherapy department decided that no rule.

A total of 41 patients received local radiotherapy. Thirty-two of them received Flank radiotherapy. Whole abdominal irradiation was needed for 9 patients. All of them, stage III FH except 2 patients with focal anaplasia. All patients received 10.8 Gy/6 fractions.

Twenty two patients have started radiotherapy within 2 weeks from the operation (53.6%), 9 patients started within 2 to 4 weeks from the operation, and 10 cases after 1 month. No documented complications from radiotherapy.

All stage I and II FH patients (*n* = 44) received EE4A protocol (two-drug regimen). All stage III patients received DD4A (*n* = 38) except 8 patients upgraded to Regimen I being blastemal predominant, but pathology revision during the study revealed that only 4 of them met the proper criteria for blastemal predominance, and it was reflected on toxicity.

### Treatment-related toxicities

There were 67 episodes of fever neutropenia experienced by 29 patients of the 92 cases studied (31.5%). Sixty-four episodes were grade 3, only 3 episodes were grade 4 according to CTCAE version 4, and one patient died out of septicemia. Five patients developed vincristine-induced ptosis during treatment (5.5%). Three patients had grade 2 toxicity, and 2 patients had grade 3. The average time of ptosis total improvement was 35 days (range from 21 days to 49 days). Two patients developed cardiotoxicity, one patient had grade 3 toxicity, and the second one had grade 4.

### Survival analysis

**Fig. 9:**
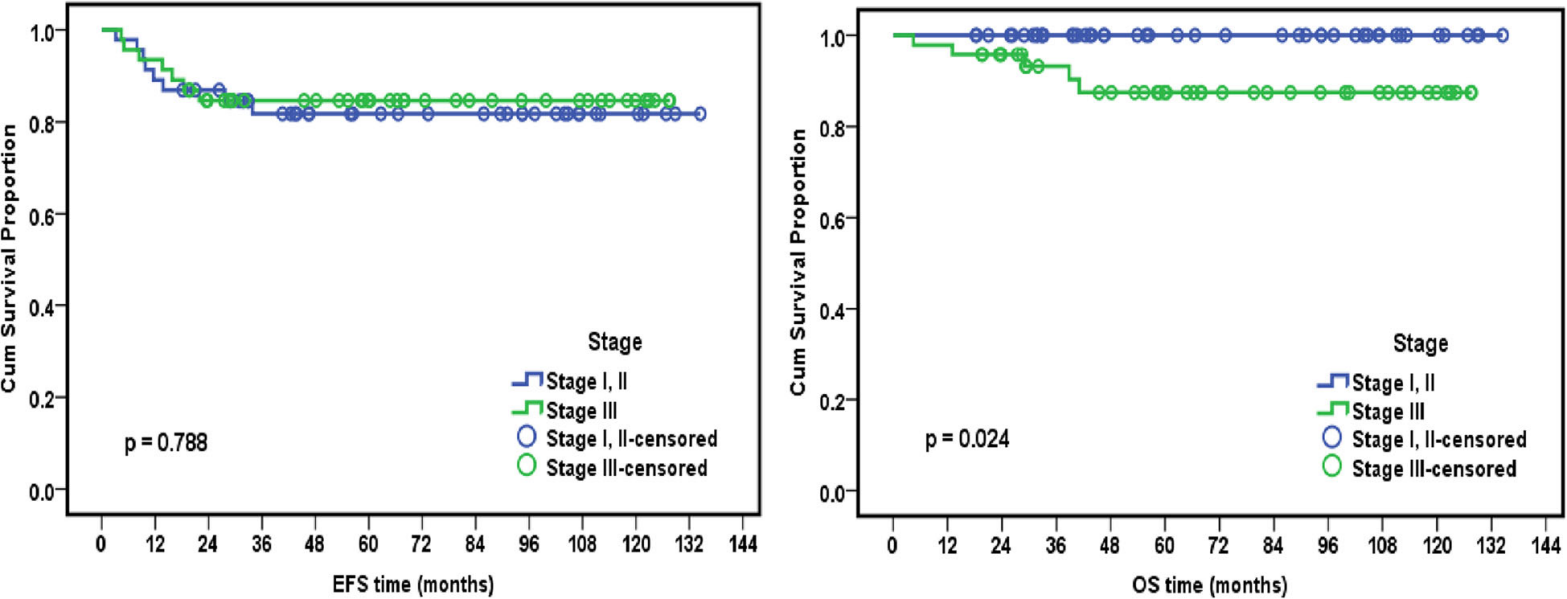
EFS and OS of different disease stages

**Fig. 10:**
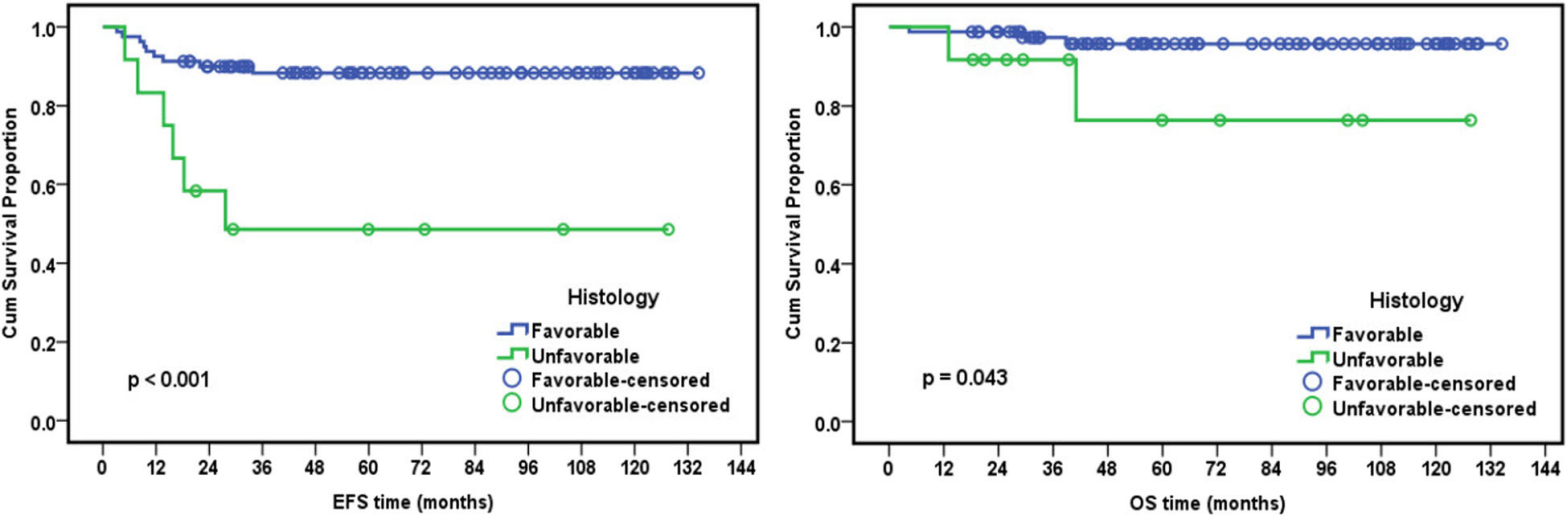
EFS and OS of cases with unfavorable histology (including cases with nuclear unrest) vs. cases with favorable histology

At a median follow-up period of 61.5 months (range 4.4 to 134.5 months), the cumulative 5-year overall survival was 94.6% and event-free survival was 83.7% with 5 deaths at the end of study period (Tables 4, 5 and 6) (Figs. 9 and 10).

**Table 4:** Event-free survival EFS and overall survival OS and their relation to the prognostic factors

	No.	No. of events	5-year EFS (%)	*p* value	No. of deaths	5-year OS (%)	*p* value
Whole group	92	15	83.7%		5	94.6%	
Gender							
Male	62	13	79.0%		4	93.5%	
Female	30	2	93.3%	0.089	1	96.6%	0.575
Age groups							
< 2 years	25	4	84.0%		0	100%	
> = 2 years	67	11	83.6%	0.949	5	92.5%	0.148
Nephrectomy							
Upfront	63	12	81.0%		2	96.8%	
Delayed	29	3	89.7%	0.337	3	89.7%	0.139
LN sampling							
Yes	49	9	81.6%		3	93.9%	
No	43	6	86.0%	0.589	2	95.3%	0.796
Stage							
Stage I and II	46	8	82.6%		0	100%	
Stage III	46	7	84.8%	0.788	5	89.1%	*0.024*
Histology							
Favorable (including nuclear unrest)	84	12	85.7%		3	96.4%	
Unfavorable	8	3	62.5%	0.073	2	75.0%	*0.009*
Histology							
Favorable	80	9	88.8%		3	96.3%	
Unfavorable (including nuclear unrest)	12	6	50.0%	< 0.001	2	83.3%	*0.043*
Stage and histology (favorable including nuclear unrest)							
Stage I/II favorable	44	8	81.8%		0	100%	
Stage III favorable	40	4	90.0%	0.304	3	92.5%	0.063
Stage I/II unfavorable	2	0	100%		0	100%	
Stage III unfavorable	6	3	50%	*	2	66.7%	*
Stage and histology (unfavorable including nuclear unrest)							
Stage I/II favorable	41	5	87.8%		0	100%	
Stage III favorable	39	4	89.7%	0.791	3	92.3%	0.065
Stage I/II unfavorable	5	3	40.0%		0	100%	
Stage III unfavorable	7	3	57.1%	0.563	2	71.4%	0.345

*No *p* value because of small number of cases within subgroups

**Table 5:** Event-free survival EFS and overall survival OS of stage III subtypes

	No.	No. of events	5-year EFS (%)	*p* value	No of deaths	5-year OS (%)	*p* value
Whole group	46	7	84.8%		5	89.1%	
Lymph node							
Negative	39	6	84.6%		5	87.2%	
Positive	7	1	85.7%	0.898	0	100%	0.285
Gross residual							
No	44	5	88.6%		4	90.9%	
Yes	2	2	0%	*	1	50%	*
Surgical margin							
Negative	42	7	83.3%		5	88.1%	
Positive	4	0	100%	*	0	100%	*
Spillage							
No	37	5	86.5%		3	91.9%	
Yes	9	2	77.7%	0.538	2	77.8%	0.271
IVC infiltration							
No	43	7	83.7%		5	88.4%	
Yes	3	0	100%	*	0	100%	*
Number of factors to be considered stage III							
0–2 factors	40	7	82.5%		5	87.5%	
> = 3 factors	6	0	100%	*0.291*	0	100%	0.364
Timing to start radiotherapy							
2 weeks	22	2	90.9%		2	90.9%	
= > 2 weeks	17	3	82.4%	*0.398*	2	88.2%	0.692
= < 1 month	31	3	90.3%		2	93.5%	
> 1 month	8	2	75.0%	*0.198*	2	75.0%	0.148

*No *p* value because of small number of cases within subgroups

**Table 6:** Time and site of relapses and deaths

No.	Stage	Gender	Age	Site of relapse			Time	Final status
				Local	Distant	Both		
1	1	Male	1 year	Local			4 months	2nd CR
2	1	Male	1 year		Lung		27 months	2nd CR
3	1	Male	4 year		Lung		22 months	2nd CR
4	1	Male	2.5 years			Local, liver	4 months	2nd CR
5	2	Male	2.5 years	Local			9 months	2nd CR
6	2	Male	1.5 years	Local			2 months	2nd CR
7	2	Male	5.5 year			Local, lung	7 months	2nd relapse
8	2	Male	1 year			Local, lung	On ttt	2nd CR
9	3	Female	5 years	Local			10 months	2nd CR
10	3	Male	5 years		Lung		6 months	2nd CR
11	3	Male	5.5 years		Lung		13 months	Died
12	3	Male	4 years		Lung		On ttt	Died
13	3	Male	2		Liver		1 month	Died
14	3	Female	4 years		Liver		4 months	Died
15	3	Male	4.5 years	Treatment-related mortality: sepsis			On ttt	Died

*ttt* treatment, *m* month

## Discussion

There are two main approaches developing a multidisciplinary treatment plan for Wilms’ tumor cases. Our institution uses the COG approach with some modifications with most of the cases underwent upfront nephrectomy and starting chemotherapy according to the surgical staging and histopathological assessment.

Intraoperative spillage percentage in our center was 8.6%, and this is similar to the percentage happened among the patients enrolled in COG AREN03B2 (11.9%) [14]. Spillage was higher in upfront nephrectomies (9.5%) than in delayed ones (6.8%).

The study showed higher incidence of advanced stages of the tumor than their average incidence in developed countries. Stage III represents 36.5% of patients (46 patients). This is similar to the percentage reported from another study that was conducted in the similar period at the Children Cancer Hospital of Egypt (CCHE) which reported a 36.7% of their patients with stage III disease [15] (Table 7).

**Table 7:** Studies discussing Wilms’ tumor survival in different centers in Egypt

Study	Results
(Zaghloul, Hussein, & El Koutbey, 1994) [16]	112 cases have been treated at NCI during the period 1979–1989 10-year OS of stage I, II, and III patients with favorable histology was 94 ± 6%, 86 ± 8%, and 71 ± 8%, respectively.
(Abd El-Aal, Habib, & Mishrif, 2005) [17]	62 cases have been treated at the pediatric unit of Kasr El-Aini center of radiation oncology and nuclear Medicine (NEMROCK) from January 1994 to January 2001. Stages I, II, and III + IV + V with favorable histology had a 4-year overall survival of 82.3, 56, and 41%, respectively.
(Naguib et al., 2008) [18]	53 cases have been treated at NCI between 2002 and 2004. 2-year OS of stages I, II, and III was 100, 100, and 61.2%, respectively.
(Salama & Kamel, 2011) [9]	65 cases have been treated at NCI between 2001 and 2008. 3-year RFS and OS for stage I, II and III were 75.4 and 77.8%, respectively.
(Elmagd Salem, Kinoshita, Abdelkhader, Hamza, & Ali, 2013) [19]	79 patients treated at the South Egypt Cancer Institute from 2002 to 2009 according to the SIOP protocol. The overall 5-year survival rate of 84% with a 5-year stage-related survival was as follows: I = 92%, II = 80%, III = 50% 33 patients treated at the Pediatric Surgery Department of Assiut University Hospital from 2000 to 2009 according to the NWTS protocol. The overall 5-year survival rate was 77% and the 5-year stage-related survival was as follows: I = 85%, II = 75%, III = 52%.
(El-­-ayadi et al., 2014) [15]	98 cases have been treated at NCI and CCHE from 2010 to 2011 3-year OS for stage I/II and stage III was 95.7% and 94.7% respectively.

In our study, 7 patients of stage III did not receive radiotherapy as indicated. They had lower EFS and OS without statistical significance, mostly due to small number of patients. The group who received radiotherapy within 2 weeks from surgical resection had better EFS and OS than that who received radiotherapy after 2 weeks. The difference became more obvious if the cutoff was 1 month from the operation, but none of these survival differences has statistical significance.

The analysis of patients enrolled in National Wilms’ Tumor Study (NWTS)-3 and NWTS-4 showed that the delay of more than 10 days did not significantly affect flank or abdominal tumor recurrence. The 8-year flank tumor recurrence rate was 1.9% for the group that received radiotherapy in less than 10 days from the operation and 1.2% for the group that received radiotherapy 10 days or more from the operation. The study recommended to give the radiotherapy within 14 days from the operation as most of the delay concentrated near 10 days, and that hindered the appearance of significant difference between the 2 groups [20].

The tumor histology has significantly impacted survival as well as tumor stage. In our study, there were 4 patients with anaplastic features, 4 patients had blastemal predominance, and 4 patients had nuclear unrest.

By the end of this study, 5 patients died out of the 92 patients studied, and 5 patients had stage III disease. Despite having less EFS in stages I and II than in stage III (81.8% vs. 83.7%), all relapsing patients with initially stage I or II disease were alive at the end of this study with 100% OS in comparison to 88.4% OS of stage III. This means that despite occurrence of relapses in stage I and II patients, they are still salvageable.

In our study, 8 out 46 stage I and II cases experienced relapse (3 patients local relapse, 2 patients distant, and 3 patients both local and distant). The higher incidence of events in early stages in our study could raise the suspicion of under staging some patients especially that lymph node sampling has been done in only 39.1% of stages 1 and II. Seven out of 46 cases of stage III Wilms’ tumor experienced an event during our study (1 local relapse, 5 distant relapses, and the last case died out of sepsis). Five out of the 7 patients died during the study. Relapsed patients after receiving three-drug regimen and local irradiation have poorer outcome [21].

The patients with nuclear unrest behavior were poorer than patients with favorable histology. Three out of 4 patients had experienced a relapse, and the fourth patient received intensive chemotherapy as it was mistakenly considered as blastemal-predominant case and is in complete remission.

The OS for unfavorable histology patients (*n* = 12) whether we considered patients with nuclear unrest as unfavorable cases or not was significantly lower than the OS of favorable patients. The EFS of the 12 patients was also lower than patients with favorable histology. The 3 patients with nuclear unrest who relapsed responded to second line and in second complete remission at the end of our study. A study comparing nuclear unrest patients with favorable histology and anaplastic patients showed that despite having a higher relapsing rate (22.2%) than the favorable histology group (12.2%), the 5-year cumulative incidence of death was not significantly higher than that in the favorable group (11.1 ± 6.2% for nuclear unrest group, and 9 ± 2.6%) [8].

Forty-seven percent of patients in our study did not have LN sampling during nephrectomy (32.6% of stage III cases and 60.8% of stage I and II patients). This is four times the percentage published among the patients enrolled in the NWTS-4 and NWTS-5 studies (12.5%) [5]. CT detected only 40% of positive LN in our study. Intraoperative gross assessment of LNs is not reliable enough, and failure to sample LN was associated with higher rates of relapse [22].

In our study, among the 15 patients who experienced relapse, 9 of them had LN sampling and 4 out of 9 had unfavorable histology (including nuclear unrest). The other 6 patients did not have LN sampling, with 2 of them having unfavorable histology. With larger number of cases, a correlation between failure to sample LN and relapse could be established, especially with having other factors impacting the relapse rate as disease stage and histology.

Five out of 49 patients who had LN sampling had at least one LN positive (10.2%). There were 2 other patients considered during treatment as LN positive upon the results of initial CTs only because they had significant lymphadenopathy at CTs; one patient did not have LN sampling at nephrectomy, and the other patient had delayed nephrectomy after resolution of lymphadenopathy as a response from preoperative chemotherapy.

A study discussing the LN involvement in patients with WT from the National Cancer Data Base (1985–2001) found 535 out of 2083 sampled patients had positive node (25.7%) [23]. Six hundred sixteen patients (18.1%) had positive LN out of 3409 cases enrolled in the NWTS-4 and NWTS-5 studies. The percentage of patients with positive LN increased with number of LNs sampled. The percentage had reached a plateau at 7 or more LN sampled (28%) [5].

The median number of LN sampled in the patients who underwent nephrectomy (3 LNs) (range 1 to 33 LNs) was lower than this plateau, and this could explain the lower incidence of LN positivity in our study (10.2%). The median LNs sampled in our study was similar to the old plateau published by Godzinski et al. [24] that demonstrated that the likelihood of LN positivity did not increase when more than 3 nodes were sampled [5].

In our study, stage III has been categorized into subtypes (positive LN, gross residual, microscopic residual, biopsy, IVC thrombus, and spillage) hoping to identify risk factors with poorer outcomes, but due to the small number of patients and smaller number of events (only 7 out of 46 patients experienced an event), none of the factors had a statistically significant impact on survival. A study published in 2013 tried to assess the impact of each criterion on survival of stage III cases enrolled in NWTS-5. None of the factors alone had a statistically significant poorer outcome, and only the group of patients who had positive LN and microscopic residual experienced lower outcome. The group with gross residual was supposed to have worse results than the group with microscopic residual, but apparently due to the lower number of this group, this difference did not manifest [25].

## Conclusions

Wilms’ tumor patients in Egypt tend to present in advanced stages, with a higher incidence of stages III and IV than developed countries. This reflects the importance of awareness among families and primary care physicians.

Despite having similar EFS, behavior of relapsing patients with initially having stage III disease was worse. This leads to significant difference in OS between stage III and stages I and II. Stage I and II relapsing patients were salvageable.

Patients with nuclear unrest had a high relapsing rate with behavior similar to unfavorable patients as anaplastic- and blastemal-predominant patients. The rate of LN sampling was much lower than the international standards with lower number of LN sampled.

More studies are needed to investigate the grades of nuclear unrest, and whether it could benefit of intensifying chemotherapy and treated as unfavorable histology.

Wilms’ tumor staging should be refined by following adequate operative data and enough LN sampling for each patient, despite the initial CT evidence or intraoperative inspection of LN.

## Limitations of the Study

1. This study was conducted retrospectively with no control on compliance to treatment or completeness of data collected.
2. The relatively small number of study patients precluded proper statistical inference for many studied variables.

## Data Availability

The datasets used and analyzed during the current study are available from the corresponding author on reasonable request.

## Abbreviations

CCHE: Children Cancer Hospital of Egypt
COG: Children’s Oncology Group
CR: Complete remission
CT: Computerized tomography
CTCAE: Common Terminology Criteria for Adverse Events
EFS: Event-free survival
FH: Favorable histology
IVC: Inferior vena cava
LN: Lymph node
LOH: Loss of heterozygosity
NCI: National Cancer Institute
NWTS: National Wilms’ Tumor Study
OS: Overall survival
RBC: Red blood corpuscle
RECIST: Response evaluation criteria in solid tumors
RFS: Relapse-free survival
SIOP: Societe Internationale D’oncologie Pediatrique
WT: Wilms’ tumor
